# Graft remodelling of hamstring ACL graft and secondary meniscal lesions are affected by tibial slope and body mass index

**DOI:** 10.1002/jeo2.70583

**Published:** 2025-12-17

**Authors:** Nicolas Vari, Emilie Bérard, Charles Andrew Slater, Thibaut Tourcher, Kenza Limam, Régis Pailhé, Hasnae Ben‐Roummane, Matthieu Ollivier, Etienne Cavaignac

**Affiliations:** ^1^ Musculoskeletal Institute Hôpital Pierre Paul Riquet, CHU Toulouse Toulouse France; ^2^ Department of Clinical Epidemiology and Public Health CERPOP, INSERM‐University of Toulouse III, Toulouse University Hospital (CHU) Toulouse France; ^3^ MedStar Health Union Memorial Hospital Baltimore Maryland USA; ^4^ Clinique Aguilera Ramsay Santé Biarritz France; ^5^ Department of Clinical Epidemiology and Public Health, Research Methodological Support Unit (USMR) Toulouse University Hospital (CHU Toulouse France; ^6^ Department of Orthopedic Surgery, Institute for Locomotion Aix‐Marseille University Marseille France; ^7^ Department of Biomechanics, APHM, CNRS, ISM, St Marguerite Hospital, Institute for Locomotion Aix‐Marseille University Marseille France

**Keywords:** graft remodelling, lateral tibial slope, risk factor, secondary meniscal lesions, SNQ

## Abstract

**Purpose:**

The purpose of this study was to evaluate which factors are associated with the remodelling of an anterior cruciate ligament graft and secondary meniscal lesions.

**Methods:**

A retrospective longitudinal study was conducted to investigate the relationship between various factors and the occurrence of graft remodelling measured with the signal to noise quotient or the appearance of secondary meniscal lesion. Data were collected prospectively and analysed retrospectively for this study. The principal endpoint was the signal‐to‐noise quotient on MRI at 1 year postoperatively. The secondary endpoint was the appearance of 1‐year secondary meniscal lesion on MRI only. The effect of the following parameters was investigated: gender, smoking status, age, body mass index, type of sport, preoperative meniscal lesion, lateral tibial slope measured on MRI, medial tibial slope measured on MRI and time from initial injury to surgery.

**Results:**

A total of 178 patients were enroled. The following factors were significantly and independently associated with the signal to noise quotient: body mass index > 25 kg/m^2^ (coefficient = 3.33 ‐ *p* < 0.001) and lateral tibial slope ≥ 1.5° (Q2‐3‐4 vs. Q1 ‐ coefficient = 2.24 ‐ *p* = 0.015). Higher preoperative body mass index (24.70 ± 3.25 vs. 22.90 ± 3.14 ‐ *p* = 0.034), medial tibial slope (odds ratio = 3.64 [95% confidence interval: 1.17–11.3] for Q3‐4 vs. Q1‐Q2 ‐ *p* = 0.019) and lateral tibial slope (odds ratio = 4.04 [1.22–13.4] for Q3‐4 vs. Q1‐Q2 – *p* = 0.016) were significantly associated with the occurrence of secondary meniscal lesions on magnetic resonance imaging 1 year after surgery.

**Conclusion:**

Having a high body mass index and an increased lateral tibial slope were significantly and independently associated with poorer graft remodelling and may lead to secondary meniscal lesions. BMI and tibial slope are key clinical factors influencing graft healing and secondary meniscal lesions after ACL reconstruction.

**Level of Evidence:**

Level III.

## Graphical abstract

This visual abstract summarises the key findings of a retrospective longitudinal study evaluating factors associated with anterior cruciate ligament (ACL) graft remodelling and secondary meniscal lesions. The study included 178 patients and assessed graft healing using the signal‐to‐noise quotient on MRI at one year postoperatively. Four main clinical factors were highlighted. First, a high body mass index (BMI > 25 kg/m²) was significantly and independently associated with poorer graft remodelling and a higher risk of secondary meniscal lesions. Second, an increased lateral tibial slope ( ≥ 1.5°) was also linked to both poor graft remodelling and the occurrence of secondary meniscal lesions. Third, secondary meniscal lesions were observed via MRI at one year and were associated with both BMI and tibial slope measurements. Fourth, MRI assessment using the signal‐to‐noise quotient provided a quantitative measure of graft healing. These findings underscore the importance of considering BMI and tibial slope in the clinical management and follow‐up of patients undergoing ACL reconstruction. The visual abstract presents these insights in a concise format with four sections, each representing one of the key factors, and concludes with a summary emphasising their clinical relevance.

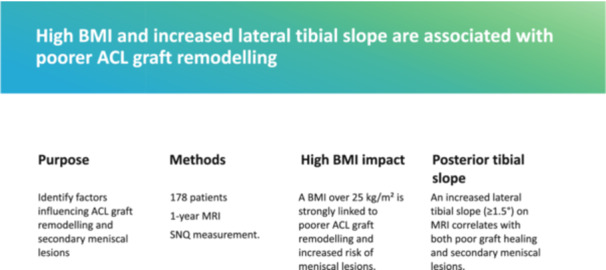

**Abbreviations**
ACL: anterior cruciate ligament
ALL: anterolateral ligament
aST: attached semitendinosus
BMI: body mass index
CI: confidence interval
IQR: interquartile range
MRI: magnetic resonance imaging
n: numbers
Q: quartile
ROI: regions of interest
SD: standard deviation
SNQ: Signal to noise quotient

## INTRODUCTION

Anterior cruciate ligament (ACL) reconstruction is a common procedure in sports medicine, yet graft failure and secondary joint damage remain significant challenges [10, 18, 26, 27, 35]. While numerous factors have been associated with the risk of graft rupture, evidence regarding the determinants of graft incorporation and remodelling remains limited and inconsistent [7, 9, 19, 22, 30].

Magnetic resonance imaging (MRI) allows non‐invasive evaluation of graft maturation, particularly through the signal‐to‐noise quotient (SNQ) [2, 3, 4, 40], which has been validated as a marker of graft remodelling and mechanical strength [11, 15, 24, 32, 41]. Variations in SNQ values have been described during the ligamentisation process [42], but the literature remains controversial regarding the influence of patient‐ and anatomy‐related factors such as body mass index (BMI) or tibial slope [39]. Moreover, the relationship between SNQ, functional outcomes, and graft failure has not been clearly established.

Secondary meniscal lesions have also been reported following ACL reconstruction and may result from instability related to impaired graft incorporation [13, 20, 33, 36]. However, the potential link between predictors of graft remodelling, changes in SNQ, and the occurrence of such secondary meniscal injuries has not been systematically investigated.

A better understanding of these mechanisms is clinically relevant, as identifying predictors of poor graft incorporation may help refine surgical indications, guide individualised rehabilitation, and reduce the risk of graft failure and secondary joint damage.

The purpose of this study was to determine, using SNQ measured on 1‐year postoperative MRI, (1) which factors influence ACL graft remodelling, and (2) whether these factors are associated with the occurrence of secondary meniscal lesions. The hypothesis was that gender, smoking status, age, BMI, type of sport, preoperative meniscal lesion, medial and lateral tibial slope, and time from injury to surgery may affect graft remodelling and the risk of secondary meniscal lesions.

## METHODS

This retrospective longitudinal study investigated the relationship between exposure factors and the occurrence of graft remodelling measured by the SNQ, on one hand, and the occurrence of secondary meniscal lesions, on the other hand. ACL reconstruction was performed using an attached semitendinosus (aST) technique + anterolateral ligament (ALL) reconstruction [31]. Patients were enroled between July 2019 and December 2022. The study was approved by the institutional review board (RnIPH 2024) and all participants provided informed consent.

### Patients

**Figure 1:**
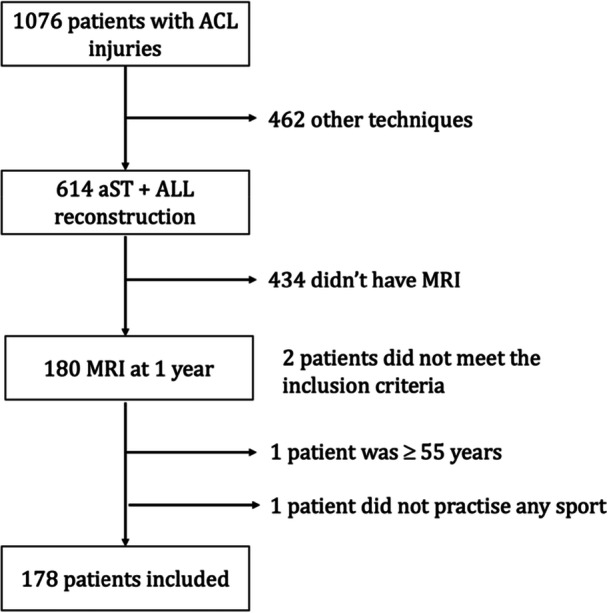
Flow‐chart of the study population. ACL, anterior cruciate ligament; ALL, anterolateral ligament; aST, attached semi‐tendinosus; MRI, magnetic resonance imaging.

During the study period, 1076 patients presented with an ACL injury were eligible for reconstruction. Of these, 614 underwent aST + ALL reconstruction and 462 underwent other techniques. 180 patients who underwent reconstruction using the aST + ALL technique had a follow‐up MRI scan 1 year after surgery (Figure 1). Only primary surgeries were considered, and revision procedures were not included in the study.

Inclusion criteria were as follows: having undergone an aST + ALL ACL reconstruction technique, willing to be reassessed at a follow‐up consultation 1 year after surgery with MRI, having undergone a primary ACL reconstruction, aged > 14 years and <55 years, healthy contralateral knee, practicing sport (recreational or high‐level activities) and managed in an university hospital in Toulouse, France.

The exclusion criteria were: cartilage lesion ≥ Outerbridge grade 2, associated fracture, previous injury to the affected knee resulting in previous surgery, and systemic disease.

### Surgical technique

**Figure 2:**
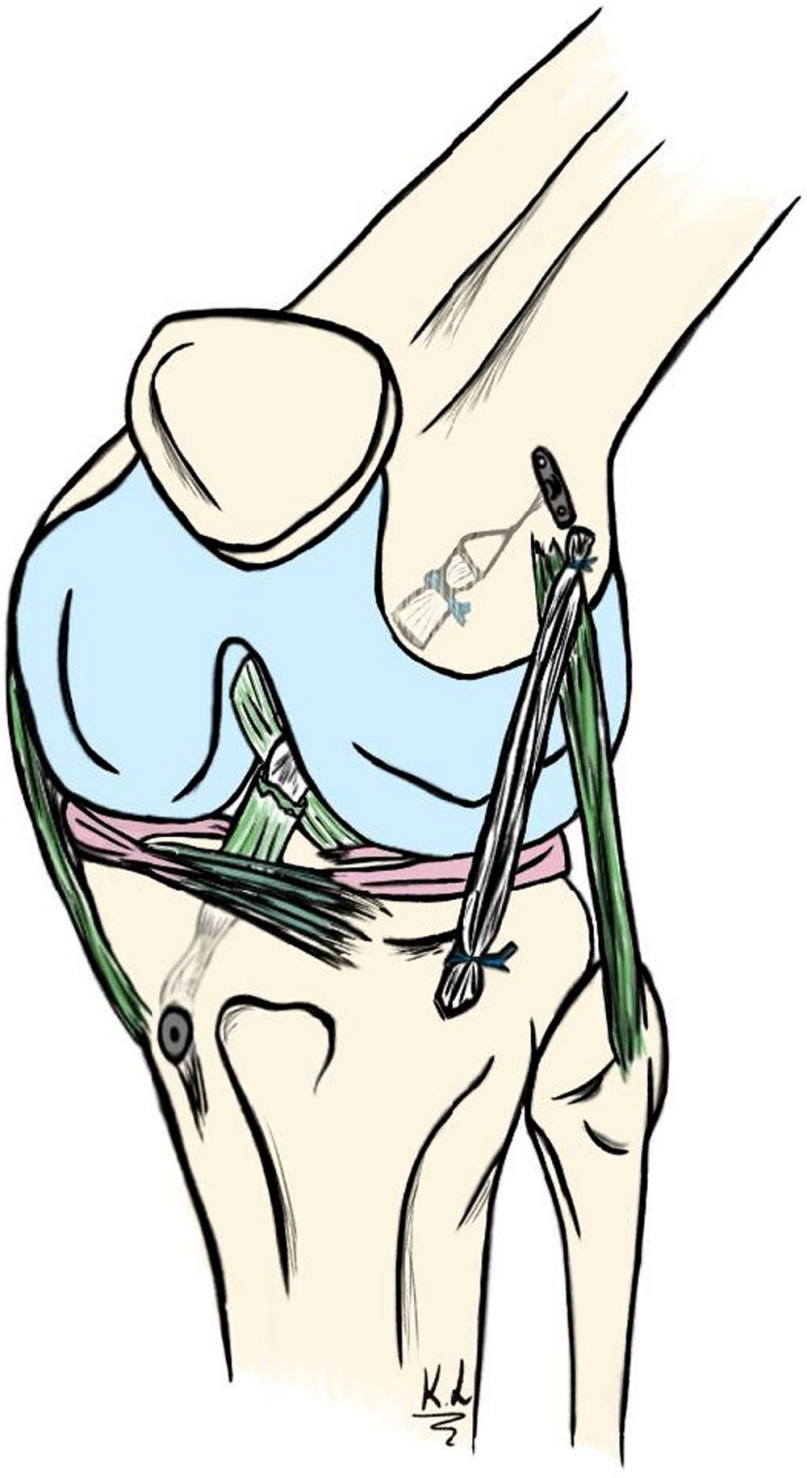
Surgical technique for anterior cruciate ligament reconstruction with the semitendinosus left attached to the tibia + reconstruction of the anterolateral ligament using the gracilis.

The patients underwent ACL reconstruction with an aST + ALL reconstruction (Figure 2). The ST tendon was left attached to its tibial insertion and then folded into four bundles to be used as a graft [31, 39, 40]. The femoral socket measured 15 mm in length. A suture button device (TightRope; Arthrex) was used for femoral fixation, and a resorbable interference screw (BioComposite Interference Screw; Arthrex) was used for tibial fixation. Anterolateral ligament reconstruction was performed using the gracilis tendon which was folded in two and passed under the fascia lata. It was then fixed in place using two anchors (Fibertack, Arthrex), one posterior to the Gerdy's tubercule and one posterior and proximal to the lateral epicondyle. The postoperative rehabilitation protocol allowed immediate weight‐bearing on the operated limb. No motion restriction or immobilisation was prescribed. Straight‐line running was initiated at 4 months postoperatively. Cutting and pivoting activities were resumed at 6 months, following satisfactory isokinetic strength testing. Return to sports was permitted from 8 months postoperatively.

### Data collection

This study examined the association of the following preoperative factors and SNQ or secondary meniscal lesions: gender (male or female), active smoking (at the time of surgery), age (in years at the time of surgery), BMI (at the time of surgery), type of sport (line, pivot, pivot‐contact), the presence of a preoperative meniscal injury (medial meniscus, lateral meniscus and/or meniscal ramp lesion), lateral and medial tibial slope (measured on MRI) and time from initial injury to surgery (weeks). Lateral and medial tibial slopes were measured using reconstructions in the sagittal plane. This was done in the centre of each tibial plateau in the frontal plane, taking as a reference the line passing through the most anterior and most posterior cartilaginous point of the plateau on the one hand, and the line passing through the centre of the tibial diaphysis on the other hand [16]. These factors have traditionally been investigated in the literature [39], and some have been associated with an increased graft rupture rate.

### Primary endpoint

The primary endpoint was the SNQ value on MRI at 1 year after surgery. During the 1‐year follow‐up visit, patients underwent an MRI of their operated knee using a 3‐T MRI machine (Magnetom Skyra; Siemens) with a 15‐channel knee coil. The following formula was used to calculate the SNQ:

$$\text{SNQ}\;=\frac{\left(\text{Graft signal}-\text{PCL signal}\right)}{\text{Background signal}}.$$

**Figure 3:**
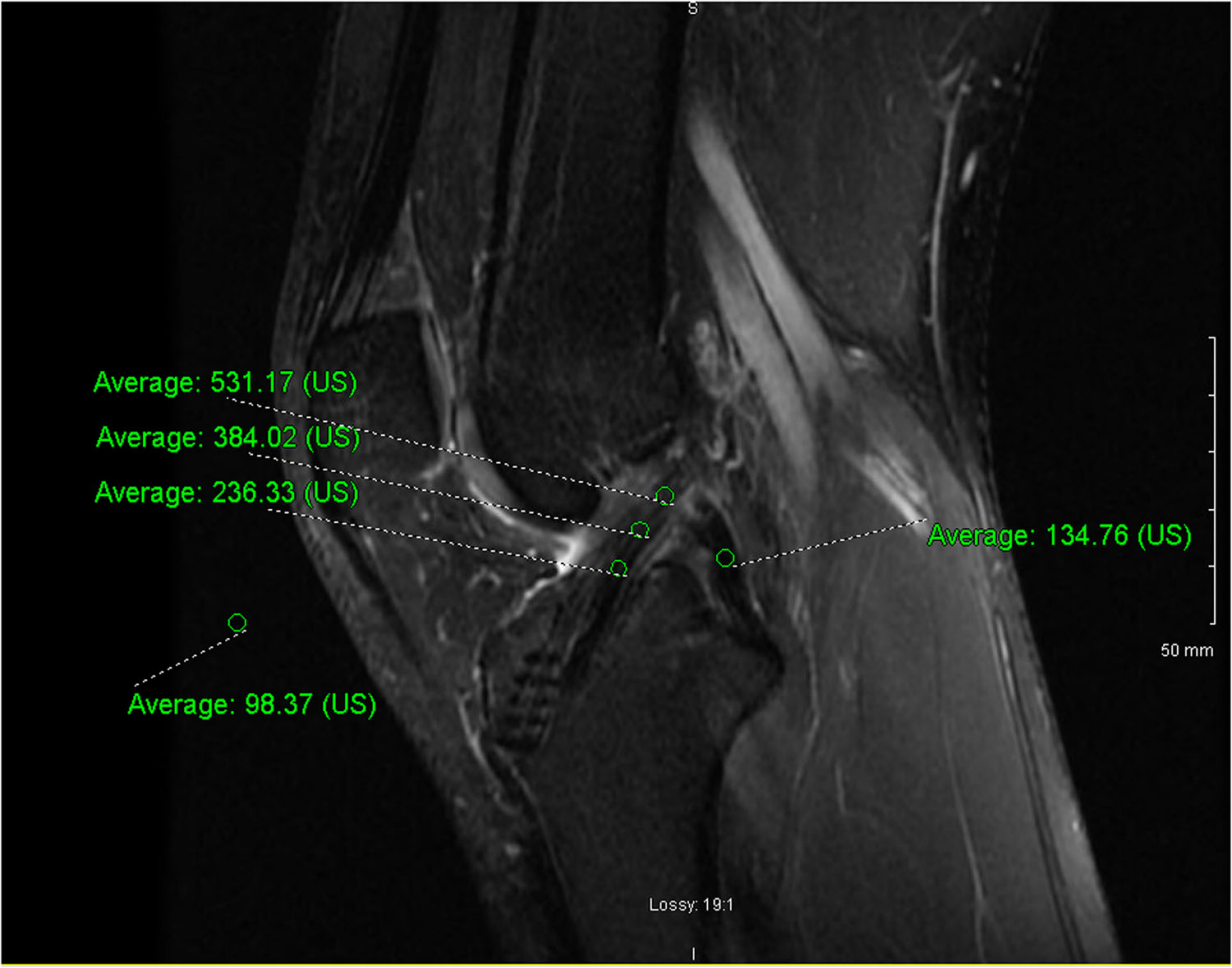
Placement of circular regions of interest (ROIs) (0.05 cm^2^) to calculate the signal‐to‐noise quotient (SNQ): 1 ROI was made 2 cm in front of the patellar tendon measuring the background signal, 3 ROIs (1 proximal, 1 middle and 1 distal) were made on the anterior cruciate ligament graft and 1 ROI was made on the posterior cruciate ligament.

The analysis was performed on a picture archiving and communication system workstation (Horizon Rad Station; McKesson). The ACL graft signal was measured on the graft in 0.05 cm^2^ circular regions of interest on T2‐weighted sagittal images where the entire intra‐articular portion of the graft was visible. Three levels (distal, middle, proximal) of the graft were measured and the mean value of the three levels was used. The PCL signal was measured in its middle portion. The background signal was measured 2 cm anterior to the patellar tendon (Figure 3). The closer the graft signal is to that of the PCL, the more advanced the graft remodelling will be and the lower the SNQ will be.

### Secondary endpoint

The secondary endpoint was the appearance of secondary meniscal lesion on MRI at 1 year after surgery. The appearance of secondary meniscal lesions was defined as the appearance, on MRI, of new lesions one year after surgery that were not present on the preoperative MRI and were not visible during surgery. These lesions were not associated with any patient‐reported traumatic event.

Patients were seen for consultation one year after surgery, following an MRI scan. A clinical examination was carried out to determine a re‐rupture, the stability of the knee, any meniscal symptoms and whether or not further surgery was necessary. Secondary meniscal lesions were assessed through clinical examination and by comparing pre‐ and postoperative MRIs, with new MRI‐detected lesions absent on the preoperative scan defined as secondary.

### Statistical analysis

All continuous variables were described using the following descriptive statistics: mean, standard deviation (SD), median, interquartile range [IQR: Q1; Q3]. Number and percentage (based on non‐missing observations) for each observed modality were reported for categorical variables.

Analysis of factors associated with SNQ was based on comparison of SNQ between groups using a Student t‐test (or Mann–Whitney test) or analysis of variance (ANOVA) (or Kruskall–Wallis test) depending on the number of groups (2 or more) and conditions of application. Variables significantly associated with SNQ in bivariate analysis (at the <0.20 threshold) were entered into a linear regression model. The final model including variables significantly (at the <0.05 threshold) and independently associated with the SNQ was obtained using a stepwise method. The nested intermediate models were compared using the likelihood ratio test. The assumptions for applying the linear regression model were checked (linearity of the risk, homoscedasticity and normality of the model's residuals) and the model's fit to the data was tested. When risk linearity was not respected, quartiles or the median threshold were used.

Analysis of the factors associated with the occurrence of secondary meniscal lesions on MRI one year after surgery was carried out using a bivariate analysis. This involved the use of the *χ* ^2^ test (or Fisher's exact test if necessary) for comparison of categorical variables, and the Student's t‐test (or Mann–Whitney test if necessary) for comparison of continuous variables. All *p*‐values were two‐sided and the significance level was < 0.05. Statistical analyses were performed using STATA® Version 18.0 software (StataCorp, College Station, TX, USA).

The priori sample size calculation was based on rules‐of‐thumb recommending between 2 and 20 subjects per variable (SPV) of a linear regression analysis. In order to analyse nine variables (gender, smoking, age, BMI, type of sport, the presence of a preoperative meniscal injury, lateral and medial tibial slope and time from initial injury to surgery), 180 subjects were included (corresponding to 20 SPV) [1, 12, 14].

## RESULTS

Of the 180 patients with a 1‐year MRI, one was over 55 years of age and one did not practise any type of sport. A total of 178 patients were analysed in this study (Figure 1).

Of the 178 patients included, 60.1% (*N* = 107) were men. The mean age at surgery was 24.32 ( ± 7.77) years and the mean 1‐year SNQ was 6.19 ( ± 5.52) (Table 1).

**Table 1:** Patient characteristics.

Variable	Statistic	Total *N* = 178
Sex		
Male	*n* (%)	107 (60.1)
Female	*n* (%)	71 (39.9)
Age at surgery (in years)	Mean (SD)	24.32 (7.77)
BMI (kg/m²)	Mean (SD)	23.58 (3.72)
Type of sport		
Line	*n* (%)	11 (6.2)
Pivot	*n* (%)	62 (34.8)
Pivot‐contact	*n* (%)	105 (59.0)
Tobacco		
No	*n* (%)	147 (82.6)
Yes	*n* (%)	31 (17.4)
Preop medial meniscus lesion	*n* (%)	13 (7.3)
Preop ramp lesion	*n* (%)	47 (26.4)	
Preop lateral meniscus lesion	*n* (%)	27 (15.2)	
Medial tibial slope (°)	Mean (SD)	5.25 (2.54)
	Median [IQR]	5.00 [4.00; 7.00]
Lateral tibial slope (°)	Mean (SD)	4.01 (3.24)
	Median [IQR]	4.00 [1.40; 6.50]
Time from initial injury to surgery in months	Mean (SD)	10.38 (25.92)
	Median [IQR]	3.53 [1.81; 7.13]
Procedure on meniscus		
None	*n* (%)	101 (56.7)
Suture repair (using hook suture for ramp lesion and all‐inside repair for other lesions)	*n* (%)	68 (38.2)
Partial meniscectomy (for instable flap lesion of medial meniscus)	*n* (%)	9 (5.1)
Graft diameter (mm)	Mean (SD)	8.74 (0.74)
Re rupture (at last follow‐up)	*n* (%)	6 (3.4)
Re operation (at last follow‐up)	*n* (%)	20 (11.2)
Revision surgery		6
Femoral anchor ablation		3
Arthrolysis		5
Partial medial meniscectomy		3
Medial meniscus repair using all‐inside procedure		2
Removal of tibial calcifications		1
Time from surgery to post‐ operative MRI in months	Mean (SD)	12.85 (2.19)
	Min–Max	10.68–26.45
1‐year postoperative medial meniscus lesion		
No	*n* (%)	148 (83.1)
De novo	*n* (%)	30 (16.9)
1‐year postoperative ramp lesion		
No	*n* (%)	178 (100)
De novo	*n* (%)	0 (0)
1‐year postoperative lateral meniscus lesion		
No	*n* (%)	163 (91.6)
De novo	*n* (%)	15 (8.4)
1‐year SNQ		
	Mean (SD)	6.19 (5.52)
	Median [IQR]	4.71 [2.65; 7.73]

Abbreviations: IQR, interquartile range; *N* or *n*, number; SD, standard deviation.

### Factors influencing graft incorporation

The relationship between the a priori selected [39] exposure factors (gender, smoking, age, BMI, type of sport, the presence of a preoperative meniscal injury, lateral and medial tibial slopes and time from initial injury to surgery) and the SNQ was investigated. In the bivariate analysis, at a threshold of 0.05, there was no significant relationship between the SNQ value and smoking habits, preoperative meniscal injury, age at surgery, medial tibial slope, lateral tibial slope and time to surgery. Female sex (*p* = 0.003), type of sport (*p* = 0.033) and BMI > 25 kg/m² (*p* = 0.012) were significantly associated with the SNQ (Table 2).

**Table 2:** Bivariate analysis of factors associated with the SNQ at postoperative 1 year.

	1‐year SNQ		
Factor	Mean	SD	*p* value
Sex			
Female	5.12	5.34	0.003
Male	6.91	5.54	
Smoking habits			
Yes	5.43	3.58	0.937
No	6.35	5.84	
Type of sport			
Line	10.02	6.84	0.033
Pivot	5.35	4.36	
Pivot‐contact	6.29	5.85	
Preop meniscal injury			
Yes	7.01	5.85	0.084
No	5.56	5.18	
Age at surgery			
≥25 years	6.49	5.65	0.750
<25 years	6.03	5.46	
BMI			
>25 kg/m^2^	8.55	7.54	0.012
≤25 kg/m^2^	5.37	4.35	
Medial tibial slope			
Q1	5.01	4.20	0.322
Q2	5.83	3.90	
Q3	6.65	6.22	
Q4	7.88	7.37	
Lateral tibial slope			
Q1	4.70	3.40	0.080
Q2	6.10	6.03	
Q3	6.36	4.78	
Q4	7.72	6.70	
Time to surgery			
Q1	6.06	6.34	0.917
Q2	6.51	5.58	
Q3	5.78	4.75	
Q4	6.44	5.44	

Abbreviations: BMI, body mass index; Q, quartile; SD, standard deviation; SNQ, signal to noise quotient.

After including all exposure factors associated with SNQ in the bivariate analysis (to the threshold of 0.20: gender, BMI, type of sport, the presence of a preoperative meniscal injury and lateral tibial slope) in a linear regression model and using a stepwise method, the multivariate analysis (Table 3) showed that the following were significantly and independently associated with poorer graft remodelling: BMI > 25 (coefficient = 3.33 ‐ *p* < 0.001) and lateral tibial slope ≥ 1.5° (Q2‐3‐4 vs. Q1 ‐ coefficient = 2.24 ‐ *p* = 0.015).

**Table 3:** Multivariate analysis of factors associated with the SNQ at postoperative 1 year.

1‐year SNQ	Coefficient	*p* value	95% CI
BMI > 25 kg/m²	3.33	<0.001	1.55–5.12
Lateral tibial slope (Q2‐Q3‐Q4 vs. Q1)	2.24	0.015	0.44–4.04

Abbreviations: CI, confidence interval; Q, quartile; SNQ, signal to noise quotient; VS, versus.

### Factors influencing the occurrence of meniscal lesions

In the bivariate analysis, at a threshold of 0.05, higher preoperative BMI (24.70 ± 3.25 vs. 22.90 ± 3.14 ‐ *p* = 0.034), medial tibial slope (odds ratio = 3.64 [95% confidence interval: 1.17–11.3] for Q3‐4 vs. Q1‐Q2) and lateral tibial slope (odds ratio = 4.04 [1.22–13.4] for Q3‐4 vs. Q1‐Q2) were significantly associated with the occurrence of secondary meniscal lesions on MRI 1 year after surgery (Table 4).

**Table 4:** Factors associated with the occurrence of secondary meniscal lesions on MRI one year after surgery.

	Occurrence of 1‐year secondary meniscal lesions			
	No	Yes	*p* value	Total
	83 (83.0)	17 (17.0)		100 (100.0)
Sex *n* (%)			0.995	
Male	44 (83.0)	9 (17.0)		53 (53.0)
Female	39 (83.0)	8 (17.0)		47 (47.0)
Age at surgery (in years)				
Mean (SD)	23.43 (7.40)	27.01 (9.72)		24.04 (7.90)
Median	22.01	22.42		22.11
IQR	[17.75; 26.00]	[20.93; 29.84]	0.145	[17.93; 26.08]
Smoking n(%)			0.751	
No	66 (83.5)	13 (16.5)		79 (79.0)
Yes	17 (81.0)	4 (19.0)		21 (21.0)
BMI (kg/m²)				
Mean (SD)	22.90 (3.14)	24.70 (3.25)	0.034	23.21 (3.21)
Median	22.22	24.44		22.48
IQR	[20.94; 24.34]	[21.80; 25.91]		[21.26; 24.69]
Type of sport *n* (%)			0.059	
Line	4 (66.7)	2 (33.3)		6 (6.0)
Pivot	32 (94.1)	2 (5.9)		34 (34.0)
Pivot‐contact	47 (78.3)	13 (21.7)		60 (60.0)
Medial tibial slope ‐ median *n* (%)			0.019	
Q1‐Q2	50 (90.9)	5 (9.1)		55 (55.0)
Q3‐Q4	33 (73.3)	12 (26.7)		45 (45.0)
Lateral tibial slope ‐ median *n* (%)			0.016	
Q1‐Q2	46 (92.0)	4 (8.0)		50 (50.0)
Q3‐Q4	37 (74.0)	13 (26.0)		50 (50.0)
Time to initial injury to surgery in months				
Mean (SD)	5.87 (9.44)	6.19 (5.97)		5.92 (8.92)
Median	2.96	3.98		3.14
IQR	[1.77; 6.67]	[2.00; 7.06]	0.289	[1.79; 6.70]

Abbreviations: IQR, interquartile range; *N* or *n*, number; SD, standard deviation.

The number of 1‐year secondary meniscal lesions did not allow multivariate analysis to be performed.

## DISCUSSION

The most important finding of this study is that high BMI and increased lateral tibial slope were significantly and independently associated with poorer graft remodelling, and both factors were also associated with the occurrence of secondary meniscal lesions at 1 year.

These results expand on the concept of biological graft failure first described by Ménétrey et al. [30], who emphasised the importance of graft vascularisation, cell proliferation, and ligamentisation [41].

Excessive BMI may impair remodelling through both mechanical overload and a systemic pro‐inflammatory state [8]. Liqiang et al. [23] reported a threshold effect of BMI on chronic inflammation, which supports this finding of impaired remodelling at higher BMI levels.

Similarly, a steeper lateral tibial slope was associated with higher SNQ values, consistent with prior studies linking posterior tibial slope to graft failure [21, 25, 42]. Tollefson et al. [38] demonstrated that increased lateral tibial slope correlates with greater anterior tibial translation, particularly in patients with extreme slopes ( > 12°). This biomechanical imbalance may compromise postoperative stability and explain the association with poorer graft remodelling observed in the present study.

In addition, high BMI and increased tibial slope were linked to secondary meniscal lesions. Schatka et al. [34] demonstrated a significant correlation between tibial slope and anterior tibial translation, while Melugin et al. [29] showed that increased posterior tibial slope augments compressive and shear forces at the posterior medial meniscus root. These mechanisms may underlie the association between tibial slope and meniscal injury in this cohort.

These findings are in line with the most recent studies [5, 28], which have consistently identified increased posterior tibial slope as a significant risk factor for graft failure after ACL reconstruction [6, 21, 37]. While some earlier investigations reported no correlation between tibial slope and clinical outcomes, more recent biomechanical and clinical data suggest that greater slope increases anterior tibial translation and shear forces on the ACL graft, thereby predisposing to re‐rupture and instability. This supports the interpretation that posterior tibial slope is associated with impaired graft remodelling and may contribute to the development of secondary meniscal lesions. The variables analysed in this study were chosen because they have traditionally been investigated as potential predictors of graft rupture or incorporation, including patient‐related (age, sex, smoking, BMI, and sport) and anatomical factors (tibial slope, meniscal status, time to surgery). This approach allows for comparison with existing literature and helps identify clinically relevant risk factors.

This study has limitations. First, it was retrospective and single‐centre, which may introduce selection bias. Second, slope measurements were performed on MRI, which is less reproducible than standard radiographs; [17] however, they were assessed by an experienced musculoskeletal radiologist. Third, SNQ analysis was performed by a single assessor, although this method has been validated as reliable and reproducible [2, 3, 22, 26, 39, 40], and the evaluation was blinded to clinical data. Finally, residual confounding may exist as not all potential risk factors could be measured.

The study also has several strengths, including the use of a uniform surgical technique by a single experienced surgeon, blinded MRI evaluation, and systematic 1‐year follow‐up, which has been shown to be sufficient to assess graft remodelling [39, 40]. The large sample size also helps to mitigate the impact of patients lost to follow‐up.

From a clinical perspective, these results emphasise the importance of considering BMI and tibial slope in preoperative risk stratification and postoperative management of ACL reconstruction patients. Identifying patients at higher risk of poor graft incorporation may allow for tailored surgical strategies, closer follow‐up, and preventive measures to reduce the risk of graft failure and secondary meniscal injury.

## CONCLUSION

Having a high body mass index and an increased lateral tibial slope were significantly and independently associated with poorer graft remodelling and may lead to secondary meniscal lesions. BMI and tibial slope are key clinical factors influencing graft healing and secondary meniscal lesions after ACL reconstruction.

## AUTHOR CONTRIBUTIONS

**Nicolas Vari**: Research. **Emilie Berard**: Statistics. **Charles A. Slater**: Research. **Thibaut Tourcher**: Research. **Kenza Limam**: Research. **Régis Pailhé**: Methodology. **Hasnae Ben Roummane**: Statistics. **Matthieu Ollivier**: Methodology. **Etienne Cavaignac**: Methodology.

## CONFLICT OF INTEREST STATEMENT

Etienne Cavaignac is a paid consultant for Arthrex, BioBank, Amplitude.

## ETHICS STATEMENT

RnIPH 2021‐64.

## Data Availability

None declared.
